# 1-Adamantylmethyl 2-amino­benzoate

**DOI:** 10.1107/S1600536810047276

**Published:** 2010-11-20

**Authors:** Zuzana Kozubková, Michal Rouchal, Marek Nečas, Robert Vícha

**Affiliations:** a Department of Chemistry, Faculty of Technology, Tomas Bata University in Zlin, Nám. T. G. Masaryka 275, Zlín,762 72, Czech Republic; b Department of Chemistry, Faculty of Science, Masaryk University in Brno, Kamenice 5, Brno-Bohunice, 625 00, Czech Republic

## Abstract

The asymmetric unit of the title compound, C_18_H_23_NO_2_, consists of two crystallographically independent mol­ecules bearing an adamantane cage consisting of three fused cyclo­hexane rings in almost ideal chair conformations, with C—C—C angles in the range 108.47 (16)–110.59 (15)°. Both aryl rings are essentially planar, the maximum deviation from the best plane being 0.0125 (19) Å. One conformer forms chains parallel to the *b* axis *via* N—H⋯O hydrogen bonds, whereas the second exhibits only an intra­molecular N—H⋯O hydrogen bond. The crystal structure is stabilized by further weak N—H⋯O and N—H⋯N inter­actions.

## Related literature

For some important biologically active compounds bearing the adamantane moiety, see: Jia *et al.* (2005 ▶); van der Schyf & Geldenhuys (2009) ▶. For the synthesis, see: Vícha *et al.* (2009 ▶).
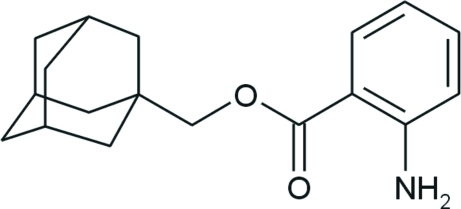


## Experimental

### 

#### Crystal data

C_18_H_23_NO_2_
*M* *_r_* = 285.37
Monoclinic, 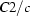

*a* = 25.8665 (19) Å
*b* = 6.4575 (4) Å
*c* = 38.6173 (8) Å
β = 106.904 (7)°
*V* = 6171.7 (6) Å^3^
*Z* = 16
Mo *K*α radiation
μ = 0.08 mm^−1^
*T* = 120 K
0.40 × 0.30 × 0.30 mm

#### Data collection

Oxford Diffraction Xcalibur diffractometer with a Sapphire2 detector
Absorption correction: multi-scan (*CrysAlis RED*; Oxford Diffraction, 2006 ▶) *T*_min_ = 0.849, *T*_max_ = 1.000
23009 measured reflections
5431 independent reflections
2752 reflections with *I* > 2σ(*I*)
*R*_int_ = 0.051

#### Refinement

*R*[*F* ^2^ > 2σ(*F* ^2^)] = 0.039
*wR*(*F* ^2^) = 0.061
*S* = 1.04
5431 reflections
395 parameters
H atoms treated by a mixture of independent and constrained refinement
Δρ_max_ = 0.21 e Å^−3^
Δρ_min_ = −0.19 e Å^−3^

### 

Data collection: *CrysAlis CCD* (Oxford Diffraction, 2006 ▶); cell refinement: *CrysAlis RED* (Oxford Diffraction, 2006 ▶); data reduction: *CrysAlis RED*; program(s) used to solve structure: *SHELXS97* (Sheldrick, 2008 ▶); program(s) used to refine structure: *SHELXL97* (Sheldrick, 2008 ▶); molecular graphics: *ORTEP-3* (Farrugia, 1997 ▶) and *Mercury* (Macrae *et al.*, 2008 ▶); software used to prepare material for publication: *SHELXL97*.

## Supplementary Material

Crystal structure: contains datablocks global, I. DOI: 10.1107/S1600536810047276/nk2072sup1.cif

Structure factors: contains datablocks I. DOI: 10.1107/S1600536810047276/nk2072Isup2.hkl

Additional supplementary materials:  crystallographic information; 3D view; checkCIF report

## Figures and Tables

**Table 1:** Hydrogen-bond geometry (Å, °)

*D*—H⋯*A*	*D*—H	H⋯*A*	*D*⋯*A*	*D*—H⋯*A*
N1—H1*B*⋯O1	0.961 (17)	2.030 (18)	2.729 (3)	128.0 (14)
N21—H21*B*⋯N1^i^	0.90 (2)	2.64 (2)	3.385 (3)	140.9 (17)
N1—H1*A*⋯O1^ii^	0.913 (19)	2.47 (2)	2.930 (2)	111.4 (15)
N1—H1*A*⋯N21^iii^	0.913 (19)	2.60 (2)	3.511 (3)	173.4 (17)
N21—H21*A*⋯O21	0.912 (18)	2.014 (19)	2.698 (3)	130.6 (16)
N21—H21*A*⋯O1^i^	0.912 (18)	2.641 (18)	3.097 (2)	111.8 (14)

Symmetry codes: (i) 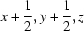; (ii) 
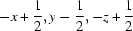
; (iii) 
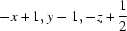
.

**Fig. 1.**
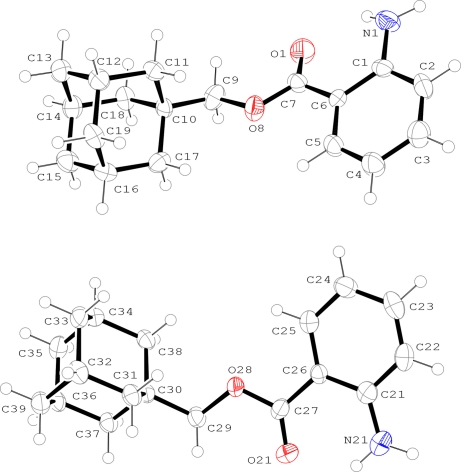
ORTEP of the asymmetric unit with atoms represented as 50% probability ellipsoids. H atoms are shown as small spheres at arbitrary radii.

**Fig. 2.**
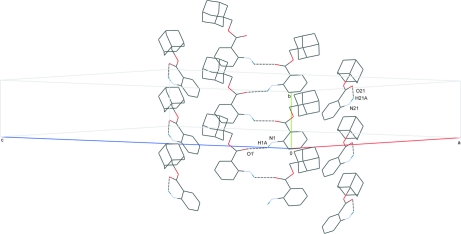
Part of the crystal structure of the title compound shows chain of one crystallographically independent conformer linked by H-bonds (dashed lines) along the b-axis. Intramolecular H-bonds N1—H1B—O1 and hydrogen atoms except for those participating in H-bonds are omitted for clarity.

## supplementary crystallographic information

### Comment

Adamantane is a polycyclic hydrocarbon isolated by Czech chemists from petroleum fraction in the year 1933. Owing to its high lipophilicity and stability, adamantane is frequently used for the modification of compounds with known biological activity. The resulting molecules can display better pharmacodynamic and/or pharmacokinetic properties, such as SQ-109 – tuberculostatic agent derived from ethambutol (*L*. Jia *et al.* 2005) or saxagliptin – type 2 diabetes medicament (van der Schyf & Geldenhuys, 2009).

The asymmetric unit of the title compound (Fig. 1) consists of two crystallographically independent molecules slightly varying in their geometries. Both benzene rings are essentially planar with maximum deviations from the best plane being 0.0080 (19) Å for atom C5 in the first molecule and 0.0125 (19) Å for atom C22 in the second one. The dihedral angle between the best planes of the benzene rings is 26.889 (6)°. The torsion angles describing arrangement of benzene ring, adamantane cage and C7—O8—C9 linker C18–C10–C9–O8, C10–C9–O8–C7, C6–C7–O8–C9 and C1–C6–C7–O1 are -177.40 (14), -152.23 (16), -177.92 (15) and 14.4 (3)°, respectively. The values of corresponding torsion angles for the second distinct conformer are 174.24 (14), 160.52 (15), 177.07 (15) and -9.1 (3)°, respectively. While one conformer forms chains *via* N—H···O H-bonds parallel to the *b*-axis, the second conformer exhibits only intramolecular N—H···O hydrogen bond (Fig. 2, Table 1).

### Experimental

The corresponding nitro ester - starting material for title compound preparation - was obtained by a procedure described previously (Vícha *et al.*, 2009). The nitro ester (100 mg, 0.3 mmol) was dissolved in 5 ml of methanol and a portion of iron powder (134 mg, 2.4 mmol) was added. Concentrated hydrochloric acid (1 ml) was added into well stirred mixture. Reaction mixture was kept under reflux until starting material disappeared. The reaction mixture was poured into 5% aqueous Na_2_CO_3_ (10 ml) and extracted with mixture of hexane/diethyl ether, 2/1, *v*/*v* several times. The collected organic layers were dried over anhydrous Na_2_SO_4_ and crude product was obtained after solvent evaporation. Column chromatography (petroleum ether/ethyl acetate, 8:1, *v*/*v*) yielded 71 mg (83%) of yellow crystalline powder. The single-crystal used for data collection was obtained by crystallization from chloroform at room temperature.

### Refinement

Carbon bound hydrogen atoms were positioned geometrically and refined as riding using standard *SHELXTL* constraints, with their *U*_iso_ set to 1.2*U*_eq_ of their parent atoms. Nitrogen bound hydrogen atoms were located in a difference Fourier map and refined isotropically.

### Crystal data

**Table:**

C_18_H_23_NO_2_	*F*(000) = 2464
*M**_r_* = 285.37	*D*_x_ = 1.229 Mg m^−^^3^
Monoclinic, *C*2/*c*	Melting point = 366–362 K
Hall symbol: -C 2yc	Mo *K*α radiation, λ = 0.71073 Å
*a* = 25.8665 (19) Å	Cell parameters from 4567 reflections
*b* = 6.4575 (4) Å	θ = 2.7–27.3°
*c* = 38.6173 (8) Å	µ = 0.08 mm^−^^1^
β = 106.904 (7)°	*T* = 120 K
*V* = 6171.7 (6) Å^3^	Block, yellow
*Z* = 16	0.40 × 0.30 × 0.30 mm

### Data collection

**Table:**

Oxford Diffraction Xcalibur diffractometer with a Sapphire2 detector	5431 independent reflections
Radiation source: Enhance (Mo) X-ray Source	2752 reflections with *I* > 2σ(*I*)
graphite	*R*_int_ = 0.051
Detector resolution: 8.4353 pixels mm^-1^	θ_max_ = 25.0°, θ_min_ = 3.2°
ω scan	*h* = −30→30
Absorption correction: multi-scan (*CrysAlis RED*; Oxford Diffraction, 2006)	*k* = −7→7
*T*_min_ = 0.849, *T*_max_ = 1.000	*l* = −38→45
23009 measured reflections	

### Refinement

**Table:**

Refinement on *F*^2^	Primary atom site location: structure-invariant direct methods
Least-squares matrix: full	Secondary atom site location: difference Fourier map
*R*[*F*^2^ > 2σ(*F*^2^)] = 0.039	Hydrogen site location: inferred from neighbouring sites
*wR*(*F*^2^) = 0.061	H atoms treated by a mixture of independent and constrained refinement
*S* = 1.04	*w* = 1/[σ^2^(*F*_o_^2^) + (0.015*P*)^2^] where *P* = (*F*_o_^2^ + 2*F*_c_^2^)/3
5431 reflections	(Δ/σ)_max_ < 0.001
395 parameters	Δρ_max_ = 0.21 e Å^−^^3^
0 restraints	Δρ_min_ = −0.19 e Å^−^^3^

### Special details

**Table:**

Geometry. All e.s.d.'s (except the e.s.d. in the dihedral angle between two l.s. planes) are estimated using the full covariance matrix. The cell e.s.d.'s are taken into account individually in the estimation of e.s.d.'s in distances, angles and torsion angles; correlations between e.s.d.'s in cell parameters are only used when they are defined by crystal symmetry. An approximate (isotropic) treatment of cell e.s.d.'s is used for estimating e.s.d.'s involving l.s. planes.
Refinement. Refinement of *F*^2^ against ALL reflections. The weighted *R*-factor *wR* and goodness of fit *S* are based on *F*^2^, conventional *R*-factors *R* are based on *F*, with *F* set to zero for negative *F*^2^. The threshold expression of *F*^2^ > 2σ(*F*^2^) is used only for calculating *R*-factors(gt) *etc*. and is not relevant to the choice of reflections for refinement. *R*-factors based on *F*^2^ are statistically about twice as large as those based on *F*, and *R*- factors based on ALL data will be even larger.

### Fractional atomic coordinates and isotropic or equivalent isotropic displacement parameters (Å^2^)

**Table:**

	*x*	*y*	*z*	*U*_iso_*/*U*_eq_	
O1	0.27207 (5)	0.3902 (2)	0.21497 (4)	0.0515 (4)	
N1	0.29950 (7)	0.0010 (4)	0.24145 (5)	0.0430 (5)	
C1	0.33939 (7)	0.0205 (3)	0.22478 (5)	0.0301 (5)	
C2	0.37972 (8)	−0.1306 (3)	0.23019 (5)	0.0372 (5)	
H2	0.3797	−0.2432	0.2460	0.045*	
C3	0.41922 (8)	−0.1190 (3)	0.21309 (5)	0.0422 (6)	
H3	0.4464	−0.2227	0.2175	0.051*	
C4	0.42012 (8)	0.0413 (3)	0.18949 (5)	0.0429 (6)	
H4	0.4476	0.0488	0.1777	0.052*	
C5	0.38052 (7)	0.1888 (3)	0.18354 (5)	0.0338 (5)	
H5	0.3805	0.2978	0.1670	0.041*	
C6	0.34023 (7)	0.1848 (3)	0.20085 (5)	0.0251 (5)	
C7	0.30047 (8)	0.3546 (3)	0.19566 (5)	0.0318 (5)	
O8	0.29882 (4)	0.4694 (2)	0.16652 (3)	0.0386 (4)	
C9	0.26212 (7)	0.6481 (3)	0.15911 (5)	0.0394 (6)	
H9A	0.2807	0.7712	0.1723	0.047*	
H9B	0.2300	0.6187	0.1674	0.047*	
C10	0.24490 (7)	0.6899 (3)	0.11885 (5)	0.0272 (5)	
C11	0.21169 (7)	0.5078 (3)	0.09824 (4)	0.0294 (5)	
H11A	0.2343	0.3814	0.1021	0.035*	
H11B	0.1805	0.4813	0.1076	0.035*	
C12	0.19154 (7)	0.5575 (3)	0.05766 (5)	0.0323 (5)	
H12	0.1700	0.4379	0.0445	0.039*	
C13	0.15625 (7)	0.7498 (3)	0.05152 (5)	0.0380 (5)	
H13A	0.1244	0.7253	0.0603	0.046*	
H13B	0.1432	0.7813	0.0253	0.046*	
C14	0.18892 (7)	0.9333 (3)	0.07187 (5)	0.0350 (5)	
H14	0.1656	1.0598	0.0679	0.042*	
C15	0.23731 (7)	0.9710 (3)	0.05761 (5)	0.0387 (5)	
H15A	0.2246	1.0045	0.0315	0.046*	
H15B	0.2586	1.0898	0.0705	0.046*	
C16	0.27263 (7)	0.7773 (3)	0.06352 (5)	0.0338 (5)	
H16	0.3043	0.8024	0.0541	0.041*	
C17	0.29290 (7)	0.7285 (3)	0.10409 (5)	0.0345 (5)	
H17A	0.3163	0.6043	0.1081	0.041*	
H17B	0.3146	0.8460	0.1171	0.041*	
C18	0.20872 (7)	0.8839 (3)	0.11237 (5)	0.0336 (5)	
H18A	0.1774	0.8598	0.1217	0.040*	
H18B	0.2294	1.0030	0.1256	0.040*	
C19	0.23996 (7)	0.5952 (3)	0.04336 (5)	0.0357 (5)	
H19A	0.2629	0.4697	0.0470	0.043*	
H19B	0.2273	0.6256	0.0171	0.043*	
O21	0.63002 (5)	0.95579 (19)	0.15600 (3)	0.0426 (4)	
N21	0.67083 (8)	0.6087 (4)	0.19276 (5)	0.0466 (6)	
C21	0.61810 (8)	0.5686 (3)	0.19077 (5)	0.0310 (5)	
C22	0.60638 (8)	0.3934 (3)	0.20847 (5)	0.0376 (5)	
H22	0.6347	0.3006	0.2199	0.045*	
C23	0.55515 (9)	0.3530 (3)	0.20973 (5)	0.0409 (6)	
H23	0.5486	0.2347	0.2225	0.049*	
C24	0.51230 (8)	0.4830 (3)	0.19257 (5)	0.0391 (5)	
H24	0.4767	0.4554	0.1936	0.047*	
C25	0.52287 (7)	0.6526 (3)	0.17406 (5)	0.0321 (5)	
H25	0.4938	0.7398	0.1616	0.039*	
C26	0.57499 (7)	0.6995 (3)	0.17309 (5)	0.0255 (5)	
C27	0.58532 (8)	0.8871 (3)	0.15428 (5)	0.0300 (5)	
O28	0.53969 (4)	0.97894 (19)	0.13458 (3)	0.0327 (3)	
C29	0.54562 (7)	1.1712 (3)	0.11641 (5)	0.0317 (5)	
H29A	0.5767	1.1605	0.1064	0.038*	
H29B	0.5525	1.2873	0.1339	0.038*	
C30	0.49414 (7)	1.2116 (3)	0.08609 (5)	0.0244 (5)	
C31	0.48638 (7)	1.0487 (3)	0.05613 (4)	0.0301 (5)	
H31A	0.5183	1.0480	0.0469	0.036*	
H31B	0.4831	0.9098	0.0661	0.036*	
C32	0.43554 (7)	1.0966 (3)	0.02513 (5)	0.0310 (5)	
H32	0.4308	0.9889	0.0059	0.037*	
C33	0.38650 (7)	1.0953 (3)	0.03946 (5)	0.0374 (6)	
H33A	0.3825	0.9568	0.0494	0.045*	
H33B	0.3535	1.1249	0.0195	0.045*	
C34	0.39332 (7)	1.2585 (3)	0.06906 (5)	0.0343 (5)	
H34	0.3611	1.2570	0.0784	0.041*	
C35	0.39899 (7)	1.4717 (3)	0.05345 (5)	0.0368 (5)	
H35A	0.4035	1.5783	0.0725	0.044*	
H35B	0.3659	1.5047	0.0337	0.044*	
C36	0.44816 (7)	1.4731 (3)	0.03881 (5)	0.0308 (5)	
H36	0.4518	1.6128	0.0286	0.037*	
C37	0.49895 (7)	1.4253 (3)	0.06989 (4)	0.0286 (5)	
H37A	0.5038	1.5326	0.0889	0.034*	
H37B	0.5310	1.4280	0.0608	0.034*	
C38	0.44432 (6)	1.2112 (3)	0.09989 (4)	0.0306 (5)	
H38A	0.4407	1.0740	0.1104	0.037*	
H38B	0.4488	1.3165	0.1192	0.037*	
C39	0.44104 (7)	1.3093 (3)	0.00931 (5)	0.0346 (5)	
H39A	0.4727	1.3104	−0.0002	0.041*	
H39B	0.4083	1.3401	−0.0109	0.041*	
H1B	0.2805 (7)	0.125 (3)	0.2443 (5)	0.054 (7)*	
H21B	0.6966 (9)	0.517 (3)	0.2037 (6)	0.094 (10)*	
H1A	0.3045 (8)	−0.098 (3)	0.2590 (5)	0.073 (9)*	
H21A	0.6781 (7)	0.722 (3)	0.1807 (5)	0.058 (8)*	

### Atomic displacement parameters (Å^2^)

**Table:**

	*U*^11^	*U*^22^	*U*^33^	*U*^12^	*U*^13^	*U*^23^
O1	0.0435 (9)	0.0695 (12)	0.0492 (10)	0.0164 (8)	0.0257 (8)	0.0112 (8)
N1	0.0432 (12)	0.0529 (16)	0.0355 (13)	−0.0100 (12)	0.0158 (10)	0.0053 (12)
C1	0.0284 (12)	0.0392 (14)	0.0219 (12)	−0.0115 (11)	0.0062 (10)	−0.0021 (11)
C2	0.0480 (14)	0.0317 (14)	0.0287 (13)	−0.0019 (12)	0.0061 (12)	0.0079 (11)
C3	0.0460 (14)	0.0404 (15)	0.0404 (14)	0.0098 (11)	0.0127 (12)	0.0029 (12)
C4	0.0452 (14)	0.0486 (16)	0.0421 (15)	0.0107 (12)	0.0240 (11)	0.0114 (13)
C5	0.0370 (13)	0.0396 (15)	0.0274 (13)	0.0035 (11)	0.0133 (11)	0.0069 (11)
C6	0.0211 (11)	0.0283 (13)	0.0240 (12)	−0.0006 (10)	0.0035 (10)	0.0013 (10)
C7	0.0334 (13)	0.0379 (14)	0.0240 (13)	−0.0071 (11)	0.0083 (11)	0.0017 (12)
O8	0.0392 (8)	0.0427 (9)	0.0329 (9)	0.0116 (7)	0.0091 (7)	0.0055 (8)
C9	0.0362 (12)	0.0390 (14)	0.0389 (14)	0.0087 (11)	0.0042 (11)	−0.0053 (11)
C10	0.0303 (12)	0.0310 (13)	0.0191 (12)	−0.0015 (10)	0.0051 (10)	0.0010 (10)
C11	0.0352 (11)	0.0279 (12)	0.0277 (12)	−0.0031 (10)	0.0131 (9)	−0.0019 (10)
C12	0.0372 (12)	0.0319 (14)	0.0252 (13)	−0.0100 (10)	0.0050 (10)	−0.0052 (10)
C13	0.0366 (12)	0.0472 (15)	0.0293 (13)	−0.0004 (12)	0.0083 (10)	0.0025 (12)
C14	0.0384 (13)	0.0295 (14)	0.0361 (14)	0.0084 (10)	0.0092 (11)	0.0024 (11)
C15	0.0450 (13)	0.0280 (13)	0.0428 (14)	−0.0057 (11)	0.0123 (11)	0.0031 (11)
C16	0.0337 (12)	0.0318 (13)	0.0392 (14)	−0.0045 (11)	0.0160 (11)	0.0013 (11)
C17	0.0271 (11)	0.0305 (13)	0.0439 (15)	0.0009 (10)	0.0070 (11)	−0.0006 (11)
C18	0.0416 (12)	0.0302 (13)	0.0296 (13)	0.0076 (10)	0.0114 (10)	0.0003 (10)
C19	0.0480 (13)	0.0329 (13)	0.0283 (13)	0.0034 (11)	0.0146 (11)	0.0038 (11)
O21	0.0267 (8)	0.0497 (10)	0.0507 (10)	−0.0001 (8)	0.0104 (7)	0.0114 (8)
N21	0.0338 (13)	0.0507 (15)	0.0521 (14)	0.0090 (12)	0.0077 (11)	0.0114 (12)
C21	0.0349 (13)	0.0371 (14)	0.0206 (12)	0.0017 (11)	0.0077 (10)	−0.0049 (11)
C22	0.0454 (14)	0.0353 (14)	0.0266 (13)	0.0074 (12)	0.0016 (11)	0.0000 (11)
C23	0.0613 (16)	0.0279 (14)	0.0324 (14)	−0.0001 (12)	0.0122 (13)	0.0038 (11)
C24	0.0408 (13)	0.0379 (14)	0.0427 (14)	−0.0015 (12)	0.0186 (11)	0.0032 (12)
C25	0.0354 (13)	0.0299 (13)	0.0298 (13)	0.0041 (10)	0.0073 (10)	0.0020 (11)
C26	0.0280 (12)	0.0264 (13)	0.0221 (12)	0.0030 (10)	0.0072 (10)	0.0013 (10)
C27	0.0255 (12)	0.0394 (14)	0.0240 (12)	0.0069 (11)	0.0055 (11)	−0.0005 (11)
O28	0.0267 (7)	0.0332 (9)	0.0345 (8)	−0.0012 (7)	0.0032 (6)	0.0119 (7)
C29	0.0330 (12)	0.0282 (13)	0.0311 (13)	−0.0032 (10)	0.0049 (10)	0.0056 (11)
C30	0.0262 (11)	0.0238 (12)	0.0217 (12)	−0.0007 (9)	0.0046 (10)	0.0022 (10)
C31	0.0364 (12)	0.0224 (12)	0.0313 (12)	−0.0003 (10)	0.0095 (10)	0.0026 (10)
C32	0.0429 (13)	0.0259 (13)	0.0221 (12)	−0.0067 (10)	0.0063 (11)	−0.0050 (10)
C33	0.0316 (12)	0.0385 (14)	0.0341 (13)	−0.0098 (10)	−0.0030 (11)	0.0100 (11)
C34	0.0256 (12)	0.0456 (15)	0.0324 (13)	0.0023 (10)	0.0095 (10)	0.0097 (12)
C35	0.0359 (12)	0.0401 (15)	0.0310 (13)	0.0075 (11)	0.0044 (10)	0.0030 (11)
C36	0.0384 (12)	0.0232 (12)	0.0299 (13)	−0.0018 (10)	0.0086 (10)	0.0068 (11)
C37	0.0327 (11)	0.0248 (13)	0.0272 (12)	−0.0028 (10)	0.0071 (10)	0.0006 (10)
C38	0.0354 (12)	0.0332 (13)	0.0240 (12)	−0.0009 (10)	0.0099 (10)	0.0031 (10)
C39	0.0357 (12)	0.0363 (14)	0.0290 (13)	−0.0015 (10)	0.0051 (10)	0.0043 (11)

### Geometric parameters (Å, °)

**Table:**

O1—C7	1.211 (2)	O21—C27	1.2222 (19)
N1—C1	1.372 (2)	N21—C21	1.368 (2)
N1—H1B	0.961 (17)	N21—H21B	0.90 (2)
N1—H1A	0.913 (19)	N21—H21A	0.912 (18)
C1—C2	1.399 (2)	C21—C22	1.400 (2)
C1—C6	1.412 (2)	C21—C26	1.408 (2)
C2—C3	1.370 (2)	C22—C23	1.365 (2)
C2—H2	0.9500	C22—H22	0.9500
C3—C4	1.384 (2)	C23—C24	1.395 (2)
C3—H3	0.9500	C23—H23	0.9500
C4—C5	1.368 (2)	C24—C25	1.378 (2)
C4—H4	0.9500	C24—H24	0.9500
C5—C6	1.392 (2)	C25—C26	1.393 (2)
C5—H5	0.9500	C25—H25	0.9500
C6—C7	1.476 (2)	C26—C27	1.476 (2)
C7—O8	1.3379 (19)	C27—O28	1.3422 (19)
O8—C9	1.4688 (18)	O28—C29	1.4562 (18)
C9—C10	1.512 (2)	C29—C30	1.518 (2)
C9—H9A	0.9900	C29—H29A	0.9900
C9—H9B	0.9900	C29—H29B	0.9900
C10—C17	1.530 (2)	C30—C38	1.531 (2)
C10—C11	1.535 (2)	C30—C31	1.533 (2)
C10—C18	1.540 (2)	C30—C37	1.535 (2)
C11—C12	1.535 (2)	C31—C32	1.531 (2)
C11—H11A	0.9900	C31—H31A	0.9900
C11—H11B	0.9900	C31—H31B	0.9900
C12—C13	1.519 (2)	C32—C33	1.524 (2)
C12—C19	1.528 (2)	C32—C39	1.526 (2)
C12—H12	1.0000	C32—H32	1.0000
C13—C14	1.533 (2)	C33—C34	1.527 (2)
C13—H13A	0.9900	C33—H33A	0.9900
C13—H13B	0.9900	C33—H33B	0.9900
C14—C15	1.526 (2)	C34—C35	1.527 (2)
C14—C18	1.531 (2)	C34—C38	1.529 (2)
C14—H14	1.0000	C34—H34	1.0000
C15—C16	1.526 (2)	C35—C36	1.535 (2)
C15—H15A	0.9900	C35—H35A	0.9900
C15—H15B	0.9900	C35—H35B	0.9900
C16—C19	1.523 (2)	C36—C39	1.526 (2)
C16—C17	1.534 (2)	C36—C37	1.531 (2)
C16—H16	1.0000	C36—H36	1.0000
C17—H17A	0.9900	C37—H37A	0.9900
C17—H17B	0.9900	C37—H37B	0.9900
C18—H18A	0.9900	C38—H38A	0.9900
C18—H18B	0.9900	C38—H38B	0.9900
C19—H19A	0.9900	C39—H39A	0.9900
C19—H19B	0.9900	C39—H39B	0.9900
			
C1—N1—H1B	117.1 (11)	C21—N21—H21B	119.5 (14)
C1—N1—H1A	116.6 (13)	C21—N21—H21A	118.2 (12)
H1B—N1—H1A	117.9 (18)	H21B—N21—H21A	122.0 (18)
N1—C1—C2	119.7 (2)	N21—C21—C22	118.7 (2)
N1—C1—C6	122.2 (2)	N21—C21—C26	123.3 (2)
C2—C1—C6	117.99 (18)	C22—C21—C26	117.94 (19)
C3—C2—C1	121.22 (19)	C23—C22—C21	121.48 (19)
C3—C2—H2	119.4	C23—C22—H22	119.3
C1—C2—H2	119.4	C21—C22—H22	119.3
C2—C3—C4	121.0 (2)	C22—C23—C24	120.88 (19)
C2—C3—H3	119.5	C22—C23—H23	119.6
C4—C3—H3	119.5	C24—C23—H23	119.6
C5—C4—C3	118.41 (19)	C25—C24—C23	118.35 (18)
C5—C4—H4	120.8	C25—C24—H24	120.8
C3—C4—H4	120.8	C23—C24—H24	120.8
C4—C5—C6	122.36 (19)	C24—C25—C26	121.76 (18)
C4—C5—H5	118.8	C24—C25—H25	119.1
C6—C5—H5	118.8	C26—C25—H25	119.1
C5—C6—C1	118.95 (18)	C25—C26—C21	119.53 (18)
C5—C6—C7	120.69 (18)	C25—C26—C27	120.45 (17)
C1—C6—C7	120.29 (18)	C21—C26—C27	120.00 (18)
O1—C7—O8	122.43 (19)	O21—C27—O28	122.17 (18)
O1—C7—C6	125.50 (19)	O21—C27—C26	125.10 (18)
O8—C7—C6	112.08 (18)	O28—C27—C26	112.73 (17)
C7—O8—C9	117.37 (15)	C27—O28—C29	116.84 (14)
O8—C9—C10	108.72 (15)	O28—C29—C30	109.06 (14)
O8—C9—H9A	109.9	O28—C29—H29A	109.9
C10—C9—H9A	109.9	C30—C29—H29A	109.9
O8—C9—H9B	109.9	O28—C29—H29B	109.9
C10—C9—H9B	109.9	C30—C29—H29B	109.9
H9A—C9—H9B	108.3	H29A—C29—H29B	108.3
C9—C10—C17	112.57 (15)	C29—C30—C38	111.68 (14)
C9—C10—C11	110.47 (15)	C29—C30—C31	110.92 (14)
C17—C10—C11	108.91 (15)	C38—C30—C31	108.83 (14)
C9—C10—C18	107.19 (15)	C29—C30—C37	108.17 (14)
C17—C10—C18	109.11 (15)	C38—C30—C37	108.64 (14)
C11—C10—C18	108.48 (14)	C31—C30—C37	108.53 (14)
C12—C11—C10	110.00 (14)	C32—C31—C30	110.24 (14)
C12—C11—H11A	109.7	C32—C31—H31A	109.6
C10—C11—H11A	109.7	C30—C31—H31A	109.6
C12—C11—H11B	109.7	C32—C31—H31B	109.6
C10—C11—H11B	109.7	C30—C31—H31B	109.6
H11A—C11—H11B	108.2	H31A—C31—H31B	108.1
C13—C12—C19	109.38 (16)	C33—C32—C39	109.54 (15)
C13—C12—C11	109.92 (15)	C33—C32—C31	109.36 (15)
C19—C12—C11	109.37 (14)	C39—C32—C31	109.63 (15)
C13—C12—H12	109.4	C33—C32—H32	109.4
C19—C12—H12	109.4	C39—C32—H32	109.4
C11—C12—H12	109.4	C31—C32—H32	109.4
C12—C13—C14	109.60 (14)	C32—C33—C34	109.93 (15)
C12—C13—H13A	109.7	C32—C33—H33A	109.7
C14—C13—H13A	109.7	C34—C33—H33A	109.7
C12—C13—H13B	109.7	C32—C33—H33B	109.7
C14—C13—H13B	109.8	C34—C33—H33B	109.7
H13A—C13—H13B	108.2	H33A—C33—H33B	108.2
C15—C14—C18	109.61 (15)	C33—C34—C35	109.28 (15)
C15—C14—C13	109.02 (15)	C33—C34—C38	109.33 (15)
C18—C14—C13	109.49 (15)	C35—C34—C38	109.11 (15)
C15—C14—H14	109.6	C33—C34—H34	109.7
C18—C14—H14	109.6	C35—C34—H34	109.7
C13—C14—H14	109.6	C38—C34—H34	109.7
C16—C15—C14	109.49 (15)	C34—C35—C36	109.62 (15)
C16—C15—H15A	109.8	C34—C35—H35A	109.7
C14—C15—H15A	109.8	C36—C35—H35A	109.7
C16—C15—H15B	109.8	C34—C35—H35B	109.7
C14—C15—H15B	109.8	C36—C35—H35B	109.7
H15A—C15—H15B	108.2	H35A—C35—H35B	108.2
C19—C16—C15	109.71 (15)	C39—C36—C37	109.59 (15)
C19—C16—C17	109.71 (15)	C39—C36—C35	109.73 (15)
C15—C16—C17	109.45 (15)	C37—C36—C35	108.90 (14)
C19—C16—H16	109.3	C39—C36—H36	109.5
C15—C16—H16	109.3	C37—C36—H36	109.5
C17—C16—H16	109.3	C35—C36—H36	109.5
C10—C17—C16	109.93 (14)	C36—C37—C30	110.36 (14)
C10—C17—H17A	109.7	C36—C37—H37A	109.6
C16—C17—H17A	109.7	C30—C37—H37A	109.6
C10—C17—H17B	109.7	C36—C37—H37B	109.6
C16—C17—H17B	109.7	C30—C37—H37B	109.6
H17A—C17—H17B	108.2	H37A—C37—H37B	108.1
C14—C18—C10	110.02 (14)	C34—C38—C30	110.57 (14)
C14—C18—H18A	109.7	C34—C38—H38A	109.5
C10—C18—H18A	109.7	C30—C38—H38A	109.5
C14—C18—H18B	109.7	C34—C38—H38B	109.5
C10—C18—H18B	109.7	C30—C38—H38B	109.5
H18A—C18—H18B	108.2	H38A—C38—H38B	108.1
C16—C19—C12	109.24 (15)	C36—C39—C32	109.27 (15)
C16—C19—H19A	109.8	C36—C39—H39A	109.8
C12—C19—H19A	109.8	C32—C39—H39A	109.8
C16—C19—H19B	109.8	C36—C39—H39B	109.8
C12—C19—H19B	109.8	C32—C39—H39B	109.8
H19A—C19—H19B	108.3	H39A—C39—H39B	108.3

### Hydrogen-bond geometry (Å, °)

**Table:**

*D*—H···*A*	*D*—H	H···*A*	*D*···*A*	*D*—H···*A*
N1—H1B···O1	0.961 (17)	2.030 (18)	2.729 (3)	128.0 (14)
N21—H21B···N1^i^	0.90 (2)	2.64 (2)	3.385 (3)	140.9 (17)
N1—H1A···O1^ii^	0.913 (19)	2.47 (2)	2.930 (2)	111.4 (15)
N1—H1A···N21^iii^	0.913 (19)	2.60 (2)	3.511 (3)	173.4 (17)
N21—H21A···O21	0.912 (18)	2.014 (19)	2.698 (3)	130.6 (16)
N21—H21A···O1^i^	0.912 (18)	2.641 (18)	3.097 (2)	111.8 (14)

Symmetry codes: (i) *x*+1/2, *y*+1/2, *z*; (ii) −*x*+1/2, *y*−1/2, −*z*+1/2; (iii) −*x*+1, *y*−1, −*z*+1/2.
